# The Ups and Downs of Plant *NLR* Expression During Pathogen Infection

**DOI:** 10.3389/fpls.2022.921148

**Published:** 2022-06-02

**Authors:** Alicia Fick, Velushka Swart, Noëlani van den Berg

**Affiliations:** ^1^Department of Biochemistry, Genetics and Microbiology, University of Pretoria, Pretoria, South Africa; ^2^Forestry and Agricultural Biotechnology Institute, University of Pretoria, Pretoria, South Africa

**Keywords:** pathogen infection, NLR, epigenetics, transcriptional regulatinon, NB-LRR, *cis* elements, NLR expression

## Abstract

Plant Nucleotide binding-Leucine rich repeat (NLR) proteins play a significant role in pathogen detection and the activation of effector-triggered immunity. NLR regulation has mainly been studied at a protein level, with large knowledge gaps remaining regarding the transcriptional control of *NLR* genes. The mis-regulation of *NLR* gene expression may lead to the inability of plants to recognize pathogen infection, lower levels of immune response activation, and ultimately plant susceptibility. This highlights the importance of understanding all aspects of NLR regulation. Three main mechanisms have been shown to control *NLR* expression: epigenetic modifications, *cis* elements which bind transcription factors, and post-transcriptional modifications. In this review, we aim to provide an overview of these mechanisms known to control *NLR* expression, and those which contribute toward successful immune responses. Furthermore, we discuss how pathogens can interfere with *NLR* expression to increase pathogen virulence. Understanding how these molecular mechanisms control *NLR* expression would contribute significantly toward building a complete picture of how plant immune responses are activated during pathogen infection—knowledge which can be applied during crop breeding programs aimed to increase resistance toward numerous plant pathogens.

## Introduction

Various pathogens, including bacteria, fungi, oomycetes, and viruses, constantly bombard plant species and may cause large crop losses in agricultural settings. Plants have in turn evolved a complex set of defense mechanisms to combat pathogen infection (Jones and Dangl, 2006). Understanding how these plant defense responses are regulated and activated during pathogen attack will accelerate crop breeding programs and may contribute to the development of transgenic crop species with the desired resistance characteristics (Wang et al., 2019). Research focused on unraveling plant immune responses has, unsurprisingly, been of particular interest for the past decade (Bezerra-Neto et al., 2020). All research studies have contributed to reveal an increasingly more complex system, with thousands of signaling molecules, receptors, and hormones, each playing a role in plant immune responses (Wan et al., 2012; Van den Berg et al., 2018; Adachi et al., 2019).

Jones and Dangl (2006) first explained plant immune responses with the well-known Zig-Zag model. This model explains that pathogens are first recognized when pathogen-associated molecular patterns (PAMPs) or damage-associated molecular patterns (DAMPs) are recognized by pattern recognition receptors (PRRs). After PAMP recognition, PRRs activate a low amplitude immune response, known as PAMP-triggered immunity (PTI). This immune response is often able to overcome infection by suppressing pathogen growth. However, some pathogens can overcome PTI responses. Plant Resistance (R) proteins may then recognize Avirulence effector (Avr) proteins, secreted by pathogens, and trigger effector-triggered immunity (ETI; Davis and Hahlbrock, 1987). A successful ETI response leads to the reactive oxygen species (ROS) and activation of the hypersensitive response (HR)—leading to localized plant cell death and the suppression of pathogen growth. When a R protein is either not present, or unable to recognize a corresponding Avr protein, effector-triggered susceptibility (ETS) is triggered, often leading to plant death (Cui et al., 2015).

This model can, however, be deceivingly simple (Naveed et al., 2020). For example, the model explains that a successful ETI response (able to overcome host-adapted pathogen attack) can only be activated when R proteins recognize a respective Avr protein. However, an increasing amount of evidence has suggested that the expression levels of *R* genes also contribute toward a successful immune response (Bradeen et al., 2009; Andam et al., 2020). When *R* gene expression is mis-regulated, the amplitude of ETI activation decreases, and an ETI response strong enough to suppress pathogen growth cannot be triggered (Umadevi and Anandaraj, 2017; Xu et al., 2018). Thus, even when a R protein is able to recognize a corresponding Avr protein, insufficient levels of this protein would lead to susceptibility. Understanding how *R* gene expression is regulated is thus the first step in untangling the mechanisms behind successful immune responses. In this review, we will focus on the regulatory mechanisms controlling *R* gene expression during pathogen infection, and how pathogens interfere and highjack these mechanisms for their own advantage.

## The Main Character: NLR Proteins

**Figure 1:**
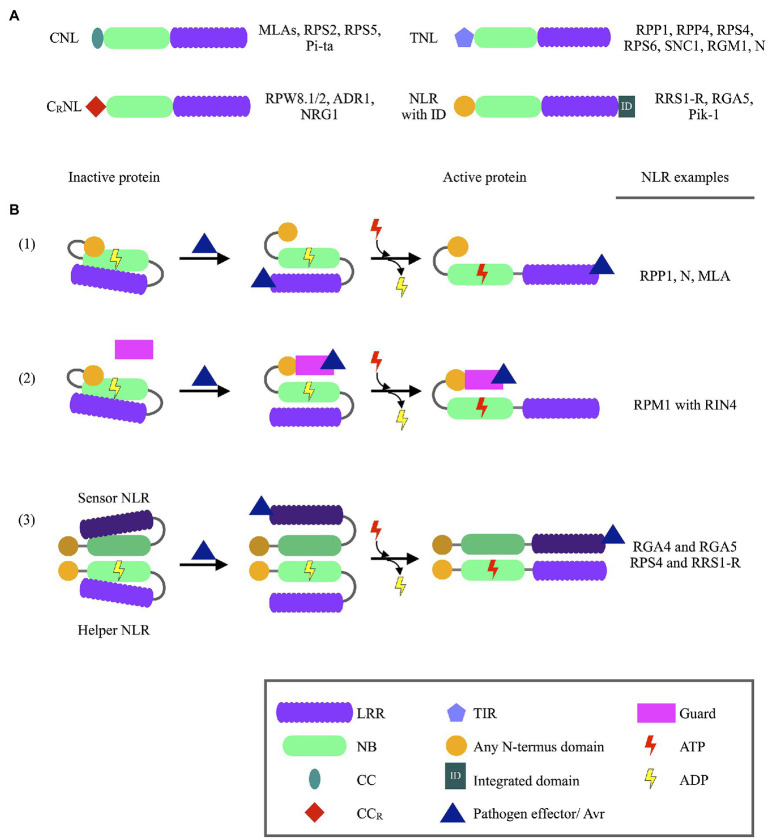
Schematic representation of NLR protein domains and NLR activation, together with NLR protein examples. **(A)** Different structures of NLR proteins identified in plants, with NLR protein examples listed on the right of each schematic. **(B)** Models of Nucleotide binding-Leucine rich repeat (NLR) protein recognition of pathogen Avirulence (Avr) proteins. (1) Direct recognition of pathogen Avr protein through binding to LRR domains of NLR proteins. Avr recognition is followed by the exchange of ADP with ATP at the Nucleotide binding (NB) domain, which activates the protein and downstream immune responses. (2) Pathogen Avr proteins may bind to guard proteins which are under the surveillance of NLR proteins. Once Avr binding is recognized the NLR protein is activated through the binding of ATP, ultimately leading to immune response activation. (3) Avr recognition by NLR dimer pairs occurs when an Avr proteins binds to a sensor NLR. Structural changes of the sensor NLR induced by the Avr activates a helper NLR, subsequently leading to immune response activation. The binding of ATP to the sensor NLR is not required for NLR function (ADP—Adenosine diphosphate; ATP—Adenosine triphosphate; CC—Coiled coil; CC_R_—Coiled coil domain with integrated RPW8 domain; LRR—Leucine rich repeat; NB—Nucleotide binding; TIR—Toll/interleukin-1 receptor).

NLR proteins, also known as NB-LRRs, constitute the largest subclass of R proteins and are characterized based on containing a Nucleotide binding (NB/NB-ARC) domain and a Leucine rich repeat (LRR) domain. NLRs can further be classified based on either having a Coiled coil (CC) domain, a CC with an integrated RPW8 domain (CC_R_), or a Toll/interleukin-1 receptor domain located at the protein’s N-terminus, termed CNLs, C_R_NLs, and TNLs, respectively (Figure 1; Takken and Goverse, 2012). These N-terminus domains are normally described to control homo- or heterodimerization events between NLRs (Xu et al., 2000; Maekawa et al., 2011). In NLR dimer pairs, one NLR often acts as a “sensor” NLR, able to recognize pathogen Avr proteins (Bonardi et al., 2011). The second NLR acts as a “helper” NLR, triggering the ETI response following activation by the sensor NLR. NB domains remain largely conserved between species and are often used during phylogenetic studies (Maiti et al., 2014). NB domains function as molecular switches for NLR proteins, determining whether the protein is in an active or inactive state. This molecular switch is controlled by ADP and ATP binding to the NB domain P-loop, with ATP binding to activate NLRs following Avr recognition (Steele et al., 2019). The LRR domain, however, is variable in length and shows large sequence variations since this domain is responsible for Avr recognition (Maule et al., 2007). The LRR domain also exerts a negative regulatory effect on the NLR, with loss of this domain leading to increased cell necrosis (Bentham et al., 2017).

The recognition of Avr proteins can either occur through direct binding to LRR domains, with the use of a guard protein, or through sensor NLR proteins (Chiang and Coaker, 2015). The use of a guard, or sensor NLRs, to recognize Avr proteins have been shown to significantly increase the variety of Avr proteins recognized by a particular NLR protein. Furthermore, many guard proteins including RIN4, are under the surveillance of multiple NLR proteins (Su et al., 2018). This increases the chances of Avr recognition and successful ETI activation. Arabidopsis RPP1 proteins serve as an example for NLR proteins which directly recognize Avr proteins (Botella et al., 1998). Some NLR proteins also contain integrated domains (ID), which may resemble Avr targets. For example, a WRKY domain was identified in the Arabidopsis RRS1-R protein which recognizes *Ralstonia solanacearum* effectors (Deslandes et al., 2002; Grund et al., 2019). *Ralstonia solanocearum* PopP2 and AvrRps4 effectors normally target WRKY transcription factors (TFs), which abolishes transcriptional activation of defense-related genes. However, when these effectors bind to RRS1-R proteins, the RRS1-R/RPP4 complex is activated and triggers defense responses (Ma et al., 2018). Thus, the WRKY ID acts as a decoy for *R. solanocearum* effectors.

## More Is Better—Sometimes: *NLR* Expression

**Figure 2:**
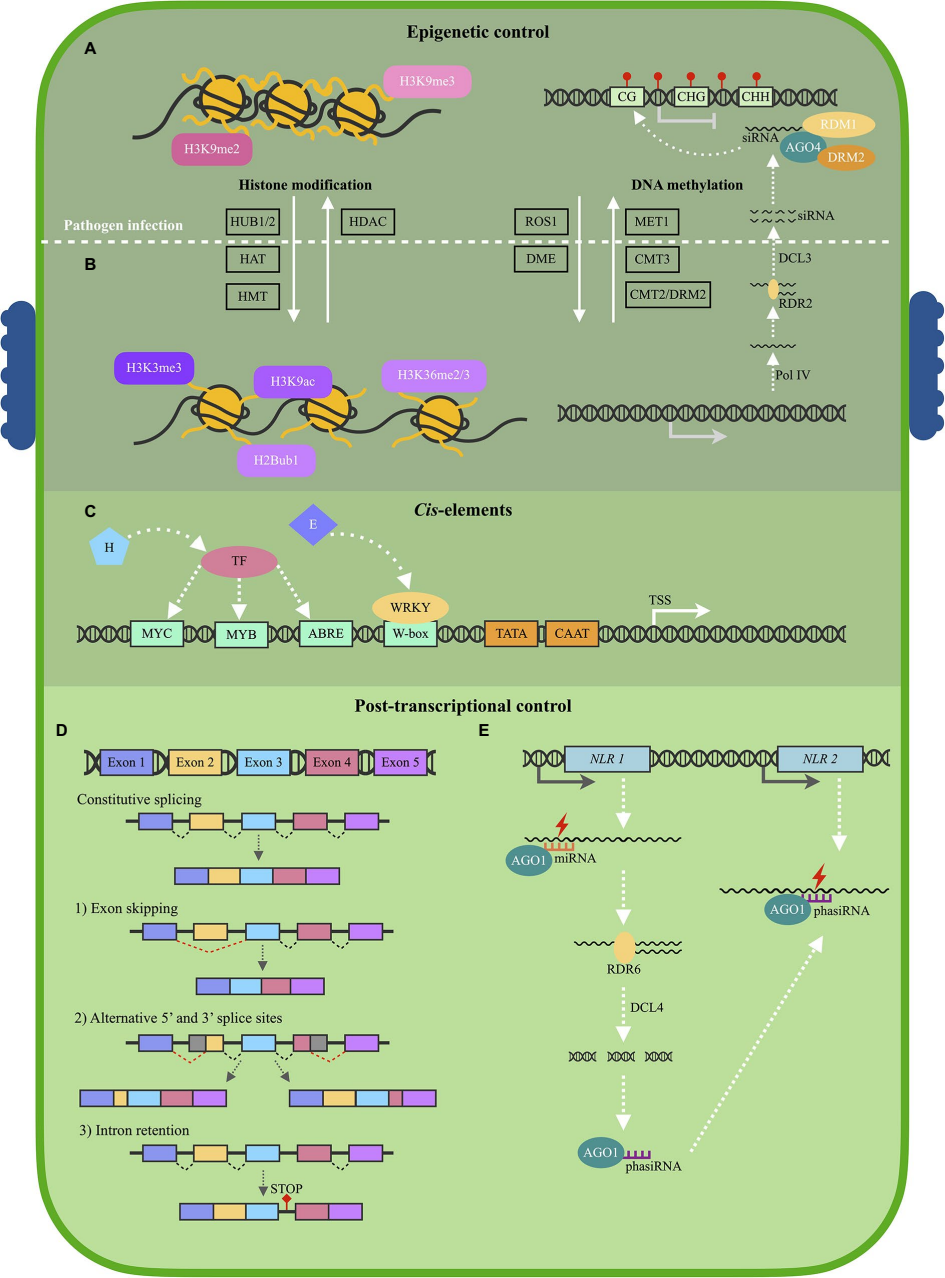
Schematic illustration of the main regulatory mechanisms of plant *NLR* expression. **(A)** Before pathogen infection, a heterochromatin structure is maintained by histone methylation marks H3K9me2 and H3K9me3, which suppresses *Nucleotide binding-Leucine rich repeat* (*NLR*) expression. Histone deacetylases (HDAC) also contribute to a heterochromatin structure. The H3K9me3 mark is also associated with DNA methylation of CG, CHG, and CHH sites, established by the RNA-directed DNA methylation pathway. *De novo* DNA methylation is guided by small interfering RNA (siRNA) in association with Argonaute 4 (AGO4), Domains rearranged methyltransferase 2 (DRM2), and RNA-dependent DNA methylation 1 (RDM1) proteins. DNA methylation is further maintained by DNA methyltransferase 1 (MET1), CMT3 (Chromomethylase 3), and CMT2/DRM2. **(B)** Following pathogen infection, a euchromatin structure is adopted which allows for the activation of *NLR* expression. Histone marks H3K3me3 and H3K36me2/3 are established by Histone methyl transferases (HMT), H3K9ac by Histone acetyltransferases (HAT), and H2Bub1 by Histone monoubiquitination 1 and 2 (HUB1/2). Repressor of silencing 1 (ROS1) and DEMETER enzymes (DME) antagonize the RNA-directed DNA methylation pathway, and lower levels of DNA methylation is observed. **(C)** A euchromatin structure allows for Transcription factors (TFs) to bind to *cis* elements within *NLR* promoter sequences located upstream from the Transcription start site (TSS). Most TFs are activated following stress hormone (H) detection, but may also be activated by pathogen effectors **(E)**. The four most common *cis* elements identified within *NLR* promoter sequences include W-boxes, ABRE, MYB, and MYC elements. **(D)** Following *NLR* expression, alternative splicing (AS) patterns may contribute to different *NLR* mRNA isoforms, and thus, different levels of NLR proteins. AS may produce mRNAs containing (1) different exons, (2) different Untranslated regions (UTRs), (3) or a retained intron which may code for a stop codon, producing a truncated NLR protein following translation. **(E)** MicroRNA (miRNA) molecules in association with AGO1 can downregulate *NLR* expression by binding to *NLR* mRNAs to either block mRNA translation or cause mRNA degradation. Phased secondary RNA (phasiRNA) molecules can also be produced when diced *NLR* mRNAs are reverse transcribed by RNA-directed RNA polymerase 6 (RDR6) and diced by Dicer-like 4 (DCL4). These phasiRNA molecules may then target more *NLR* mRNA molecules to further contribute to *NLR* suppression (DCL3—Dicer-like 3; Pol IV—RNA polymerase IV).

NLR regulation has mainly been studied on protein level, and very little is known regarding *NLR* transcriptional regulation (Yu et al., 2022). Studies focusing on the expression of *NLR* genes have highlighted the importance of proper timing and level of *NLR* expression to activate successful immune responses during pathogen attack (Liu et al., 2020a). The overexpression of *NLR* genes leads to stunted growth and Avr-independent cell death (Palma et al., 2010; Li et al., 2015). However, rice mutants in which the APIP4 *NLR* was knocked down showed increased susceptibility when infected with *Magnaporthe oryzae* (Zhang et al., 2020). Thus, enough NLR proteins need to be activated to trigger successful immune responses during pathogen infection. This indicates that *NLR* expression needs to be above a certain threshold (Bieri et al., 2004). These factors make it unsurprising to observe differences in *NLR* expression levels between resistant and susceptible plant genotypes when infected with a pathogen. For example, 22 *NLRs* were upregulated in *Raphanus sativus* resistant to *Plasmodiophora brassicae*, but not in a susceptible genotype during *P. brassicae* infection (Wang et al., 2022a). Furthermore, significant differences in *NLR* expression were observed between a partially resistant and susceptible *Persea americana* rootstocks infected with *Phytophthora cinnamomi*, especially after 6 h post-inoculation (Fick et al., 2022). The expression of *NLR* genes is regulated by three main mechanisms: (1) epigenetic mechanisms, (2) *cis* elements and TFs, and (3) post-transcriptional modifications (Figure 2; Bezerra-Neto et al., 2020). Epigenetic mechanisms include histone modifications and DNA methylation, which influence chromatin density, and subsequently the ability of TFs and transcription machinery to bind to gene promoter sequences. TFs, which bind to *cis* elements in gene promoter sequences either exert a positive or negative regulatory effect on gene transcription. Furthermore, post-transcriptional modifications include alternative splicing patterns and small RNA, which can introduce stop codons, change protein structure or cause *NLR* mRNA degradation.

### Epigenetic Control of *NLR* Genes

Epigenetic modifications regulate whether the chromatin is in a euchromatin (open) or heterochromatin (condensed) structure, thus controlling whether *NLR* transcription can be activated (Figures 2A,B). Histone modifications and DNA methylation patterns are dynamic molecular mechanisms able to change chromatin structure following pathogen infection (Zhang et al., 2018). Histone modifications have mostly been studied for Arabidopsis *NLR* genes. One histone modification often associated with transcriptional activation is the trimethylation of lysine 4 of histone H3 (H3K4me3) and is observed to regulate the expression of the Arabidopsis *RPP4* and *SNC1 NLR* genes (Kouzarides, 2007; Xia et al., 2013). This histone mark is established by the histone methyltransferase ATXR7, with the expression of both *NLRs* being reduced in *atxr7* mutants (Xia et al., 2013). Expression of *LAZ5*, another Arabidopsis *NLR* gene, is also controlled by histone methylation. Histone methyltransferase SDG8 is responsible for di- or trimethylating H3K36, activating *LAZ5* transcription (Palma et al., 2010). The di- and trimethylated H3K36 mark, is interestingly associated with alternative splicing patterns (discussed below) of *NLR* genes. H3K36me2/me3 levels were significantly higher at the 5’ UTR (untranslated region) of the *ARG1 NLR* gene in resistant *Sorghum bicolor* genotypes when infected with *Colletotrichum sublineola* (Lee et al., 2022). H3K36me2/me3 was shown to increase the expression of *ARG1* and regulate alternative splicing patterns to produce a full-length *ARG1* mRNA transcript. In the susceptible *S. bicolor* genotype, lower H3K36me2/me3 marks and expression of *ARG1* was observed, together with truncated *ARG1* mRNA. This indicates that histone modifications also control *NLR* expression in an indirect manor at post-transcriptional levels.

Histone acetylation is also associated with active transcription of *NLR* genes (Luna et al., 2012). Histone acetylation is established by histone acetyltransferases (HATs) and removed by histone deacetylases (HDACs; Bannister and Kouzarides, 2011). Acetyl groups are negatively charged, and histone acetylation would thus result in the chromatin adopting a euchromatin structure (Luna et al., 2012). One HDAC, HDA9, in association with HOS15 was shown to regulate the expression of 62 *NLR* genes in Arabidopsis, with *hda9* and *hos15* mutants showing increased *NLR* expression levels and fewer H3K9ac marks (Yang et al., 2019). Overexpression of another HDAC protein, HDA19, was also shown to enhance Arabidopsis resistance toward the necrotrophic fungus *Alternaria brassicicola* (Zhou et al., 2005). Since NLR proteins activate the HR, decreased *NLR* expression could be hypothesized to lead to increased resistance toward necrotrophic pathogens. Histone deacetylation and thus, transcriptional repression might be favorable toward certain types of pathogens. *HDA19* expression was also induced by jasmonic acid (JA) and ethylene—signaling hormones produced in response to necrotrophic pathogens (Li et al., 2019). This shows that some histone modifying proteins are activated by either biotrophic- or necrotrophic pathogens, resulting in a different immune response which would prove more suitable toward a specific pathogen. Lastly, histone ubiquitylation is also associated with *NLR* transcription. HUB1 and HUB2, both E3 ubiquitin ligases, mono-ubiquitylates H2B to H2Bub1 during *Pseudomonas syringae* pv. *tomato* DC3000 infection of Arabidopsis. H2Bub1 levels increase, leading to the subsequent increase in expression of *RPP4* and *SNC1* (Zou et al., 2014).

The H3K9me2 histone mark is associated with transcriptional repression and is seen at the first intron region of the Arabidopsis *RPP7 NLR* gene (Tsuchiya and Eulgem, 2013). This mark influences alternative polyadenylation patterns of this *NLR* mRNA, influencing RPP7 protein structure and ultimately governs resistance levels toward *Hyaloperonospora arabidopsidis* (Eulgem et al., 2007; Tsuchiya and Eulgem, 2013). The H3K9me3 mark is also functionally associated with DNA methylation. DNA methylation of cytosine (position 5; 5mC) occurs at GC, CHG, or CHH (where H is A, C, or T) sites within plants, often aimed at silencing transposable elements (TEs) frequently found within *NLR* sequences (Miyao et al., 2003; Cuerda-Gil and Slotkin, 2016). *De novo* DNA methylation is mainly controlled by the RNA-directed DNA methylation pathway (RdDM) in plants (Law and Jacobsen, 2010). Small interfering RNAs (siRNAs) are produced during the canonical RdDM pathway when double-stranded RNA is synthesized by RNA-dependent RNA polymerase 2 (RDR2) and diced by Dicer-like 3 (DCL3). This double-stranded siRNA molecule is then incorporated into Argonaute 4 (AGO4) as single-stranded siRNA. In association with the AGO4-siRNA complex, Domains rearranged methyltransferase 2 (DRM2) and RNA-dependent DNA methylation 1 (RDM1) establishes *de novo* DNA methylation (Wendte and Pikaard, 2017). Thereafter, DNA methylation is maintained by MET 1 (DNA methyltransferase 1) at CG sites, CMT3 (Chromomethylase 3) at CHG sites, and CMT2/DRM2 at CHH sites. DNA methylation defective Arabidopsis plants showed increased resistance levels toward *P. syringae* pv. *tomato* DC3000 and *H. arabidopsidis*, indicating that decreased levels of DNA methylation may lead to increased *NLR* expression and immune activation (Dowen Robert et al., 2012; López Sánchez et al., 2016).

The widespread loss of DNA methylation (hypomethylation) at TEs has been observed to occur during the activation of immune responses following pathogen infection (Annacondia et al., 2021). Demethylation of promoters leads to *cis* elements being more accessible to TFs, ultimately leading to increased *NLR* gene expression and disease resistance. In poplar trees infected with *Lonsdalea populi*, hypomethylation occurred at CH sites within promoter regions of defense-related genes (Xiao et al., 2021). Higher levels of hypomethylation were particularly noted in poplar trees with increased resistance toward *L. populi* when compared to susceptible trees. This observation suggests that hypomethylation results in increased defense-related gene expression which may ultimately lead to increased resistance levels. This could be explained by the observation that AGO4 is repressed after Arabidopsis treatment with PAMP flagellin-22 (flg22), and *Aegilops tauschii* infection with *Blumeria graminis* f. sp. *tritici* (Yu et al., 2013; Geng et al., 2019). The repression of AGO4 leads to lower levels of DNA methylation, which decreases transcriptional repression. Furthermore, ROS1 (Repressor of silencing 1) antagonizes RdDM mediated DNA methylation and promotes resistance toward *P. syringae* pv. *tomato* DC3000 (Halter et al., 2021). ROS1 has specifically been implicated in the regulation of some Arabidopsis *NLR* genes. In *ros1* mutants, four *NLR* genes showed decreased expression levels, due to active demethylation being repressed (Kong et al., 2020). ROS1 was also shown to demethylate promoter regions in which WRKY TFs bind (Halter et al., 2021). In particular, ROS1 demethylated promoter regions of *RGM1 TNL* after PTI activation by *P. syringae* pv. *tomato* DC3000 flagellin proteins. Lastly, DNA demethylation by DEMETER (DME) enzymes also contributes to enhanced resistance toward *Verticillium dahlia* and *P. syringae* pv. *tomato* infection in Arabidopsis (Zeng et al., 2021). In *dme* mutants, a hypermethylated region was associated with the *AtPRX34 TNL* gene. This gene showed lower expression levels following *P. syringae* pv. *tomato* infection in *dme* mutants when compared to wild-type plants. These results indicate that DME demethylates *NLR* sequences in response to bacterial and fungal infection.

### *Cis* Elements of *NLR* Genes

*Cis* elements of *NLR* genes remain largely unknown due to these genes having unusually large promoter sequences (Yu et al., 2022). An *NLR* promoter sequence has often been defined as the 2 kb region upstream from the *NLR* transcription start site (Figure 2C). Multiple *cis* elements are frequently identified within *NLR* promoter sequences, many being pathogen-inducible *cis* elements (Table 1). These *cis* elements may be found in different arrangements within promoter sequences, resulting in complex gene regulatory mechanisms (Wang et al., 2021). Regulatory mechanisms are further complicated by the fact that the TFs which bind to these *cis* elements either exert a positive or negative regulatory effect on gene expression. Both a positive and negative *cis*-acting element were identified within the *SNC1 NLR* gene promoter using CRISPR/Cas9 directed mutations in Arabidopsis (Yu et al., 2022). This study further identified that two other *NLR* genes, *RPP4* and *SIKIC2*, are also affected by these mutations. This may indicate that these genes share the same *cis* elements. This hypothesis is supported by the fact that plant *NLRs* are often found within gene clusters and arranged in a head-to-head configuration (Narusaka et al., 2009; Van Wersch and Li, 2019). Many of these *NLRs* are often co-expressed following infection, further suggesting that these genes might be under the control of the same promoter, or promoters with the same *cis* elements (Liang et al., 2019; Yang et al., 2021).

**Table 1:** *Cis* elements identified in promoter sequences of plant *NLR* genes.

*Cis* element	Species	Putative function	Reference
Common			
CAAT	*Pinus monticola*	Common element	Liu and Xiang, 2019 Wang et al., 2022b Rampino et al., 2017
	*Lagenaria siceraria*		
	*Triticum durum*		
TATA-box	*P. monticola*	Core element	Liu and Xiang, 2019 Wang et al., 2022b Qi et al., 2022 Rampino et al., 2017
	*L. siceraria*		
	Tomato		
	*T. durum*		
Pathogen-inducible/stress			
ABRE	Rice	Abscisic acid responsive element	Ding et al., 2020 Wang et al., 2022b Rampino et al., 2017
	*L.a siceraria*		
	*T. durum*		
AS-1 (TGACG)	Rice	Salicylic acid responsive element	Kong et al., 2018 Goyal et al., 2021 Diao et al., 2021
	*Vitis vinifera*		
	*Glycine max*		
BIHD-binding site (TGTCA)	*P. monticola*	Regulation of defense-related genes	Liu and Xiang, 2019
CGCG-box (ACGCGT)	*P. monticola*	Stress tolerance genes	Liu and Xiang, 2019
CGTCA-motif and TGACG-motif	*L. siceraria*	Methyl jasmonate responsive element	Wang et al., 2022b Rampino et al., 2017 Cui et al., 2017
	*T. durum*		
	*G. max*		
E-box (CANNTG)	Rice	Jasmonic acid responsive element	Miyamoto et al., 2012
ERE-box (ATTTCAAA)	*P. monticola*	Ethylene responsive element	Liu and Xiang, 2019 Diao et al., 2021 Wang et al., 2020 Rampino et al., 2017
	*G. max*		
	*Actinidia chinensis*		
	*T. durum*		
G-box	Rice	Regulation of defense-related genes	Kong et al., 2018 Rampino et al., 2017
	*T. durum*		
GARE-motif, P-box, and TATC-box	*L. siceraria*	Gibberellin responsive element	Wang et al., 2022b Rampino et al., 2017 Cui et al., 2017
	*T. durum*		
	*G. max*		
GCC-box (AGCCGCC)	Rice	Ethylene and pathogen responsive gene	Kong et al., 2018 Wang et al., 2021
	*Saccharum*		
	*spontaneum*		
GT1-box (GAAAAA)	*P. monticola*	Pathogen and salt-induced gene expression	Liu and Xiang, 2019
GTTGA	*Zea mays*	*Rhizoctonia solani* inducible	Li et al., 2017
H-box (CCTACCN7CT)	Rice	Regulation of defense-related genes	Kong et al., 2018
MYB recognition elements	Rice	Stress responsive elements	Kong et al., 2018 Ding et al., 2020 Wang et al., 2020
	*S. spontaneum*		
	*A. chinensis*		
Myb1-box (GTTAGTT)	*P. monticola*	Regulation of defense and drought-related genes	Liu and Xiang, 2019 Wang et al., 2022b
	*L. siceraria*		
MYC elements	Rice	Stress responsive elements	Ding et al., 2020
STRE	Rice	Stress responsive elements	Ding et al., 2020
TATTT	*Z. mays*	*Rhizoctonia solani* inducible	Li et al., 2017
TC-rich repeats	*V. vinifera*	Stress responsive element	Goyal et al., 2021 Wang et al., 2022b Cui et al., 2017
	*L. siceraria*		
	*G. max*		
TCA element	*V. vinifera*	Salicylic acid responsive element	Goyal et al., 2021 Cui et al., 2017
	*G. max*		
TGA element	*V. vinifera*	Auxin responsive element	Goyal et al., 2021 Wang et al., 2022b
	*L. siceraria*		
W-box (TTTGACY)	*P. monticola*	Regulation of defense-related genes	Liu and Xiang, 2019 Wang et al., 2021 Wang et al., 2020
	*V. vinifera*		
	Arabidopsis		
	*S. spontaneum*		
	*A. chinensis*		
Other (Growth/development)			
ACACNNG	*P. monticola*	Abscisic acid induced gene expression	Liu and Xiang, 2019
ARR1-binding site (AGATT)	*P. monticola*	Cytokinin responsive gene	Liu and Xiang, 2019
MADS-box/ CArG-motif (CCW6GG)	*P. monticola*	Regulation of plant flowering time and vernalization genes	Liu and Xiang, 2019
Circadian motif (CAAN4ATC)	*P. monticola*	Circadian gene expression	Liu and Xiang, 2019
NtBBF1 binding site (ACTTTA)	*P. monticola*	Tissue-specific expression and auxin induction	Liu and Xiang, 2019
SRE (TTATCC)	*P.s monticola*	Activation of axillary bud outgrowth	Liu and Xiang, 2019
T-box (ACTTTG)	*P. monticola*	Light activated element	Liu and Xiang, 2019
WUS-binding site (TTAATGG)	*P. monticola*	Establishment and maintenance of stem cells in shoot and floral meristems	Liu and Xiang, 2019
W-box	*Malus domestica*	Sorbitol inducible element	Meng et al., 2018

It is important to remember that the abundance of TFs and certain arrangements of *cis* elements also influence gene expression levels, and the simple binding of a specific TF does not necessarily activate gene expression (Hoang et al., 2017). Thus, the identification of *NLR cis* elements alone cannot be used to predict the level of *NLR* expression, or when transcription will be activated. In tomato plants, a single nucleotide difference was identified in the promoter region of the *Sl5R-1 NLR* gene when compared between a Tomato spotted wilt virus (TSWV) resistant and susceptible plant (Qi et al., 2022). This single nucleotide deletion in resistant tomato plants resulted in a new TF binding site to be formed, which increases *Sl5R-1* expression and subsequent resistance. Importantly, *cis* elements are not the only regulatory sequences to control *NLR* expression—the tobacco *N TNL* contains two introns which contribute to increased expression levels of this gene during Tobacco mosaic virus (TMV) infection (Ikeda et al., 2021). Transient expression of the *N* gene without these introns showed lower levels of expression.

*Cis* elements identified most often in *NLR* promoter sequences include W-boxes, ABRE, MYB, and MYC elements (Mohr et al., 2010; Ding et al., 2020). W-boxes bind WRKY TFs, which is a large, diverse group of zinc finger TFs (Babu et al., 2006). These TFs are mostly activated by pathogen infection, effectors, and stress hormones, such as salicylic acid (SA) and JA. Following activation, a subset of WRKYs trigger the expression of PTI and ETI-related proteins, and the synthesis of stress hormones (Chen et al., 2019). Interestingly, an apple (*Malus domestica*) *NLR* gene, *MdNLR16*, is under the control of the MdWRKY79 TF which is responsive to sorbitol levels (Meng et al., 2018). Higher sorbitol levels lead to increased *MdNLR16* expression and subsequently enhanced resistance levels toward *Alternaria alternata*. ABRE elements are abscisic acid responsive elements which are recognized by bZIP proteins (Hobo et al., 1999). A single ABRE element, however, is not able to activate transcription, instead multiple elements are needed for transcriptional activation (Shen et al., 1996). Lastly, MYB and MYC elements have been shown to activate gene transcription in response to both abiotic and biotic stressors (Feng et al., 2013; Fang et al., 2018; Wu et al., 2019). *NLR cis* element identification studies have thus shown that the TFs controlling *NLR* expression is mostly activated by abiotic and biotic stress. *Cis* element studies may then also be used to identify putative NLR function. For example, in *Pinus monticola* Douglas ex D. Don (Western white pine trees), *cis* element identification of the *PmTNL2* gene suggested that this NLR might be important for both plant immune responses, as well as growth and development (Liu and Xiang, 2019).

### Post-transcriptional Modifications of NLRs

Alternative splicing (AS) contributes significantly toward the diversity of the *NLR* transcriptome and NLR proteome—altering levels of different mRNA isoforms in response to developmental and environmental conditions (Kelemen et al., 2013). AS can result in *NLR* mRNAs to contain different exons, 5′- and 3′ untranslated regions, and introns which may introduce stop codons resulting in truncated NLR proteins (Figure 2D). The example used most often for AS of *NLR* genes is the N TNL protein associated with resistance toward TMV. The *N* gene produces either a short *N* mRNA (*N_S_*) or a long *N* mRNA (*N_L_*; Erickson et al., 1999). *N_L_* contains an exon which encodes a stop codon, resulting in a truncated protein. *N_S_* however is translated into a complete protein. Both these proteins are expressed during TMV infection and needed for full TMV resistance. One rice *CNL*, *Pi-ta*, produces up to 11 different protein isoforms as a result of AS (Costanzo and Jia, 2009). In response to *M. oryzae* infection, a resistant rice genotype showed increased expression levels of a Pi-ta protein with a C-terminus thioredoxin domain, when compared to a susceptible genotype.

In both barley and wheat, AS was seen to regulate whether and which IDs were present in NLR proteins in response to different experimental conditions (Halterman et al., 2003; Andersen et al., 2020). Different IDs may influence where NLR proteins localize to in the plant cell or may even cause the NLR protein to act as a decoy target for Avr proteins (Yang et al., 2014). It is worth noting that AS may also have an impact on proteins guarded by NLRs, suggesting that AS may regulate NLR activity in an indirect manner. For example, a NLR Rpi-vnt1.1 guards the GLYK (Glycerate 3-kinase) protein in potato (Gao et al., 2020). A truncated isoform of *GLYK*, which does not contain a chloroplast transit peptide-encoding sequence, is expressed in dark conditions, and cannot be recognized by a *Phytophthora infestans* Avr protein AVRvnt1. Thus, Rpi-vnt1.1 cannot activate immune responses. In light conditions, the full-length *GLYK* mRNA is expressed, and this protein isoform binds to the Avr during infection, leading to Rpi-vnt1.1 being activated to trigger ETI.

*NLR* repression can be regulated at a post-transcriptional level using siRNAs and micro RNAs (miRNAs; Figure 2E). miRNAs are non-coding RNAs between 20 and 24 nucleotides in length. They are encoded by *miRNA* genes, which are transcribed by RNA polymerase II to produce a long, primary miRNA (Xie et al., 2005). After processing, a precursor miRNA (pre-miRNA) is produced which forms a hairpin structure with a self-complementary stem loop. This pre-miRNA molecule is diced by DCL1 or DCL4, and produces a 22 nucleotide double-stranded miRNA, which is exported to the cytoplasm (Sun et al., 2019). An RNA-induced silencing complex (RISC) is then formed when the mature miRNA binds to an AGO1 protein. miRNAs guide AGO1 proteins to target mRNAs, either resulting in endonucleolytic cleavage and degradation or inhibition of translation. The P-loop domain, important for ATP binding and NLR protein activation, is a common target for miRNAs (Zhai et al., 2011; Fei et al., 2015).

*NLR* mRNA cleavage by miRNAs may also produce phasiRNAs (phased secondary small interfering RNAs), which then target and degrade other mRNAs with the same sequence (Liu et al., 2020b). Three *Medicago truncatula* miRNA families target mRNA transcripts of 74 *NLRs*, leading to the production of phasiRNAs which suppress the expression of 324 *NLR* genes (Zhai et al., 2011). Liu et al. (2014) showed that the barley miR9863 family targets *MLA1 CNL* transcripts, with the resulting phasiRNAs also leading to *MLA1* mRNAs being degraded. The authors suspect that this pathway prevents immune responses from being overloaded, and thus, *NLR* downregulation may have a positive effect on plant resistance levels. In Arabidopsis *miR472* knock-down mutants, increased resistance levels were observed toward *P. syringae*, and reduced resistance levels were observed when this miRNA was overexpressed (Boccara et al., 2014). This presents an interesting method of pathogen control—transient expression of miRNA targets in host plants may increase resistance levels toward various pathogens. In tomato, transient expression of short tandem target mimic RNAs increased resistance levels toward *P. infestans* and *P. syringae* (Canto-Pastor et al., 2019). These mimic RNAs acted as targets for miR482/211b, which resulted in increased *NLR* expression and enhanced disease resistance.

## Oh No You Do Not: How Pathogens Interfere With *NLR* Expression

With multiple proteins contributing to the regulation of *NLR* expression, comes multiple opportunities for pathogen interference. Despite this, very few cases are documented in which pathogen Avr proteins influence *NLR* expression. However, Avr targets remain largely unknown, and it is yet to be discovered how *NLR* regulation is hijacked by pathogens (Wu et al., 2022). An average of 32% of Avr proteins from bacteria, fungi, and oomycetes localize in the plant cell nucleus, indicating that these Avr proteins may interfere with *NLR* transcription (Khan et al., 2018). Two cytoplasmic effectors from *M. oryzae*, MoHTR1, and MoHTR2, bind to effector binding elements (EBE) in rice gene promoters and function as transcription repressors (Kim et al., 2020). These EBEs were present in many defense-related gene promoters, and the binding of these effectors led to significant transcription reprogramming. Transient expression of *MoHTR1* and *MoHTR2* in rice not only led to increased susceptibility toward *M. oryzae*, but also to *Xanthomonas oryzae* pv. *oryzae* and *Cochliobolus miyabeanus*. It remains unclear whether these effectors bind host repressor proteins, or whether they interfere with the binding of transcription activators. A *Melampsora larici-populina* effector, Mlp124478, also interferes with the transcription of WRKY TFs which indirectly inhibits the activation of defense-related gene expression, including the *RPP8 NLR* (Ahmed et al., 2018). Some pathogen Avr proteins also interfere with the synthesis of stress hormones. SA metabolism is inhibited by *Phytophthora sojae* PsIsc1 and *Verticillium dahliae* VdIsc1 enzymatic effectors, which redirects the precursor molecule of SA from the plastid into the cytosol (Liu et al., 2014). The metabolism of SA decreases and thus SA-mediated immune responses cannot be activated. Since some TFs which bind to *NLR cis* elements are activated by SA, lower SA levels may disrupt the activation of *NLR* expression (Goyal et al., 2021).

From another perspective, the suppression of *NLR* expression may not be the ultimate goal of the pathogen. *Phytophthora* species are hemibiotrophic oomycetes, which switch from a biotrophic to necrotrophic phase during infection (Zuluaga et al., 2016). During the necrotrophic phase, increased *NLR* expression may be beneficial to the pathogen since NLRs activate the HR and thus, plant cell death. *P. sojae* RxLR effectors PSR1 and PSR2 suppress plant RNA silencing by interfering with the miRNA synthesis pathway, which increased susceptibility in *Nicotiana benthamiana* (Qiao et al., 2013). This may lead to higher NLR levels and activation of HR, resulting in a more favorable environment for necrotrophic pathogens. This hypothesis is further supported by the fact that PSR2 is only expressed during the later stages of infection, when *P. sojae* switches to a necrotrophic stage (Qiao et al., 2013). Furthermore, the *V. dahliae* VdSSR1 protein was shown to interfere with the nuclear exportation of AGO1-miRNA complexes in *N. benthamiana* (Zhu et al., 2022). Decreased AGO1-miRNA exportation would subsequently lead to decreased suppression of *NLR* expression, which may contribute to the observed increased susceptibility in transgenic plants expressing *VdSSR1* at higher levels. However, VdSSR1 expression data is needed to indicate whether this protein is only expressed during the necrotrophic stage of this hemibiotrophic fungus. Lastly, a necrotrophic fungus, *Botrytis cinerea*, is able to translocate siRNAs into plant cells and may redirect host siRNA machinery (Weiberg et al., 2013). *B. cinerea* siRNAs were associated with AGO1 proteins during infection of Arabidopsis, indicating that *B. cinerea* may hijack RISC to increase virulence (Ellendorff et al., 2009). It would be interesting to investigate whether these siRNA molecules cause siRNA-directed cleavage and degradation of *NLR* mRNA transcripts (Qiao et al., 2021). It may also be of interest to investigate whether pathogen-derived siRNAs influence DNA methylation patterns during infection.

## Conclusion

NLR proteins play a significant role in activating plant immune responses during pathogen attack. The mis-regulation of NLR-encoding genes considerably impairs the plant’s ability to detect pathogen Avr proteins, which ultimately leads to susceptibility. Thus, a comprehensive understanding of *NLR* gene regulation is of particular interest. Unfortunately, NLR protein regulation has mainly been studied on a post-translational level, with a large knowledge gap remaining regarding the transcriptional- and post-transcriptional regulation of these proteins. Identifying epigenetic marks, and *cis* elements which control *NLR* expression in response to pathogen attack provides the first step in unraveling these complex regulatory mechanisms. These mechanisms can further be compared between susceptible and resistant plant genotypes to understand the factors which contribute to a successful immune response. Furthermore, investigating how pathogens interfere with these mechanisms would provide much needed insight into plant–pathogen interactions. Ultimately, knowledge in these areas may be used during plant breeding programs which aim to produce genotypes with increased resistance toward a variety of pathogens. These mechanisms can also be used to drive the expression of trans-*NLR* genes in genetically modified crops, with the goal of increasing resistance toward both biotic and abiotic stresses.

## Author Contributions

AF conceptualized, drafted, and reviewed the manuscript. VS and NB reviewed and assisted in the drafting of the manuscript. All authors contributed to the article and approved the submitted version.

## Funding

Funding was generously provided by the Hans Merensky Foundation.

## Conflict of Interest

The authors declare that the research was conducted in the absence of any commercial or financial relationships that could be construed as a potential conflict of interest.

## Publisher’s Note

All claims expressed in this article are solely those of the authors and do not necessarily represent those of their affiliated organizations, or those of the publisher, the editors and the reviewers. Any product that may be evaluated in this article, or claim that may be made by its manufacturer, is not guaranteed or endorsed by the publisher.
